# Femoral Nerve Injury as a Complication of Percutaneous Simple Renal Cyst Sclerotherapy with Ethanol: A Case Report

**DOI:** 10.1155/2012/589108

**Published:** 2012-03-26

**Authors:** Alireza Ashraf, Mohammad Yasin Karami, Aida Amanat

**Affiliations:** ^1^Department of Physical Medicine and Rehabilitation, Shiraz University of Medical Sciences, Shiraz, Iran; ^2^Student Research Committee, Jahrom University of Medical Sciences, Jahrom, Iran; ^3^Trauma Research Center, Shiraz University of Medical Sciences, Shiraz, Iran

## Abstract

Simple renal cysts are benign, common, and often asymptomatic disease in old age, sometimes treated with ethanol sclerotherapy. We report a case of iatrogenic femoral nerve injury following percutaneous injection of ethanol into a renal cyst under sedation. The percutaneous injection was guided by sonography. At the end of the procedure, the cyst ruptured so the patient progressed to loss of consciousness due to alcohol intoxication. Ethanol was damaged to the femoral nerve, so patient was developed with limping, numbness, and weakness in anteromedial aspect of the right thigh. To the best of our knowledge, this is the first report of femoral nerve injury caused by percutaneous simple renal cyst sclerotherapy with ethanol. This rare event has not been previously described, Physicians should be aware of the possibility of this complication.

## 1. Introduction

Simple renal cyst is a benign, common, and often asymptomatic disease in old age. Treatment of simple renal cyst is indicated when the cyst is sufficiently large and cause complaints or when associated with complications [1].

The main indication of the percutaneous treatment of simple renal cysts is ipsilateral flank pain, hydronephrosis, and hypertension.

Sclerotherapy with ethanol has been suggested as a simple, minimally invasive, cost-effective, well-tolerated, and highly effective method for symptomatic simple renal cysts treatment [2].

In the review of some studies, there is no major complication for this procedure, except a case of coma caused by ethanol sclerotherapy [3]. Also, the potential adverse effect of ethanol in nerve damage has been shown in most studies [4].

It is thought that gynecologic surgery such as abdominal hysterectomy is one of the most common causes of femoral nerve injury. Also other causes of femoral nerve injury are retroperitoneal bleeding after femoral vein or artery puncture, cardiac angiography, central line placement, retroperitoneal fibrosis, injury during femoral nerve block, diabetic amyotrophy, infection, cancer, pregnancy, radiation, acute stretch injury due to a fall or other trauma, hemorrhage after a fall or other trauma, spontaneous hemorrhage generally due to anticoagulant therapy, idiopathic, and hypertrophic mononeuropathy. Electrodiagnostic studies (nerve conduction studies and electromyography) are the “gold standard” to confirm the presence of a femoral nerve injury. These should be performed no earlier than 3 to 4 weeks after the injury [5].

## 2. Case Report

A 61-year-old man, a known case of coronary artery disease that 11 years ago coronary artery bypass graft (CABG) was done for him, was referred by an urologist to our rehabilitation clinic with complaint of burning pain, limping, numbness, and weakness in anteromedial aspect of the right thigh after right simple renal cyst ethanol sclerotherapy 5 months prior of our visit. The patient stated that an urologist tried to ablate a huge simple renal cyst with ethanol (300 milliliter, %96) under sedation; during injection of last volume of ethanol and aspiration of cyst fluid the patient sensed a general burning pain in the right side of lumbar region that eventually progressed to loss of consciousness. One day after this procedure, he was discharged with drowsiness condition and postalcohol intoxication mood without significant improvement of his problems (burning pain, limping, numbness, and weakness in anteromedial aspect of the right thigh).

In physical examination, he had some degree of limping of the right lower extremity. The patient had difficulty in stair climbing. The lower extremity inspection did not have significant finding. Palpation over the inguinal ligament exacerbated the patient's symptoms. There was tenderness over the thigh and groin as well and pain exacerbated with hip extension. Manual muscle testing of the quadriceps femoris and iliopsoas muscles showed very mild weakness (4+/5). However, adductor group was normal. Right knee deep tendon reflex was absent. Achilles' deep tendon reflex was normal. Also right foot, knee, and hip range of motion were normal. He had difficulty to do supine active straight leg raising (SLR) test. In sensory examination, the numbness was extending into the anterolateral and medial of the leg.

**Figure 1:**
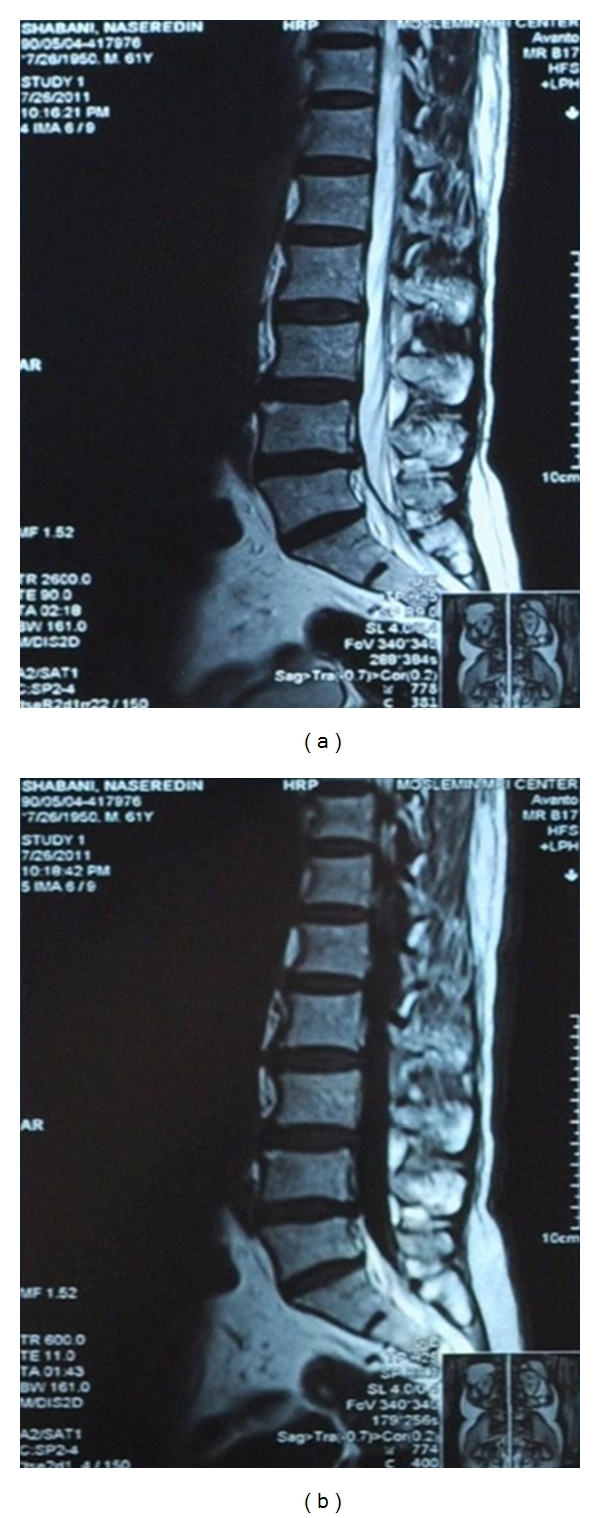
On sagittal T1-weighted (a) and T2-weighted (b) lumbar MR images lumbar degenerative joint change and lumbar disc dehydration with decreased lumbar lordosis is seen.

**Figure 2:**
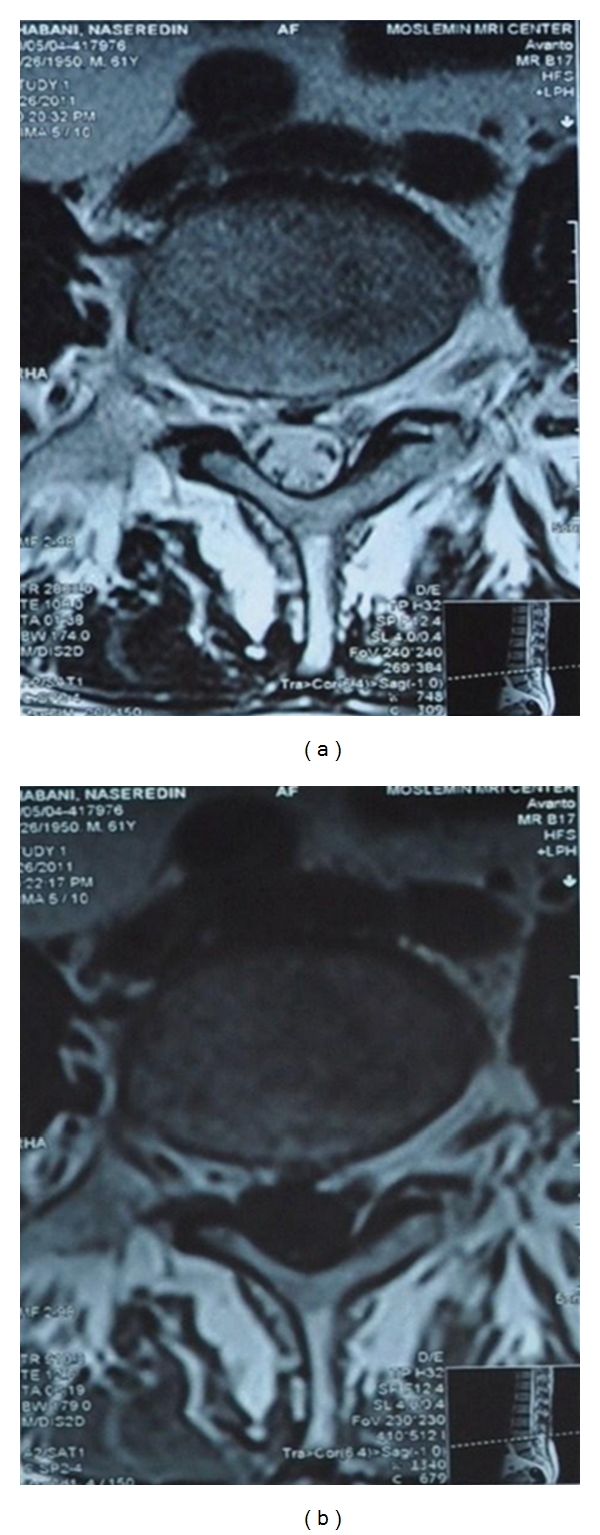
Axial T1- (a) and T2-weighted (b) lumbar MR images showed central protrusion of L4-L5 disc with slight nerve roots compression bilaterally.

For evaluation of lumbosacral region, lumbosacral magnetic resonance imaging (MRI) and electrodiagnosis were performed. T1-weighted and T2-weighted sagittal and T2-weighted axial images were obtained using a 1.5 tesla MRI system (Avanto Siemens machine). MRI showed some degree of lumbar degenerative joint change and lumbar disc dehydration with decreased lumbar lordosis that were compatible with the patient's age and a central protrusion of L4-L5 disc with slight nerve roots compression bilaterally (Figure 1). There was no evidence of spinal stenosis. Visible cord, conus medullaris, cauda equine, and para vertebral soft tissue were normal (Figure 2).

Electromyography (EMG) and nerve conduction study (NCS) confirmed right femoral nerve injury (sensory more than motor part). Therefore, nonsurgical therapy including 10 sessions of physical therapy with hot pack, electrical stimulation, sensory desensitization exercise, and quadriceps strengthening exercise was started.

Pregabalin tablet (75 mg) twice daily was prescribed as well. After nonsurgical therapy followup, the response was modest. Therefore, acupuncture was suggested as well. But no improvement was seen after complete acupuncture therapy, so it may confirm a poor prognosis of his problems.

## 3. Discussion

Renal cysts are very common abnormalities, mainly in the aging individuals over 50 years about 50% incidence, and the cyst size also increases with age [6]. The cysts remain asymptomatic, and many are found incidentally in renal ultrasonography (US), and generally these lesions do not require invasive treatment. Rarely, some cysts may cause complications such as pain, hematuria, or a nonspecific gastrointestinal manifestation, infection, hypertension, obstruction of the proximal urinary tract, and even renal failure in some complicated cases [6, 7]. Medical analgesics fail to relieve abdominal pain, so decompressing procedures for the cysts may be necessary, including percutaneous aspiration followed by instillation of sclerosing agents [8]. In several studies it was reported that simple aspiration and sclerotherapy are minimally invasive procedures, and ethanol therapy had been widely used for the treatment of symptomatic renal cysts [6, 9]. There is no study that reports a significant major complication for ethanol sclerotherapy. However, various minor ethanol-related complications have been noted such as pain, fever, and systemic reactions such as drunken state or shock; moreover, the recurrence rate has been reported to be 32% after a single-session alcohol sclerotherapy [6].

The lumbar plexus is derived from the anterior rami of the L1 through L4 nerve roots. These rami pass downward and laterally along the psoas major muscle where they eventually form the plexus, while within the psoas they divide into anterior and posterior branches. The posterior branches of anterior rami, L2–L4, become the femoral nerve, which exits from the lateral aspect of the psoas, traveling through the iliacus and under the inguinal ligament to the anterior thigh. The lumbar plexus also gives off the lateral femoral cutaneous nerve of the thigh, iliohypogastric, ilioinguinal, and genitofemoral nerves [10].

The kidneys are anterior to the diaphragm, the medial and lateral arcuate ligaments, psoas major, quadratus lumborum and the aponeurotic tendon of transversus abdominis, the subcostal vessels and subcostal, iliohypogastric, and ilioinguinal nerves.

According to our study, the author guessed that femoral nerve injury may occur due to kidney surroundings and proximity with lumbar plexus, nerve roots, iliopsoas muscle, and its blood supply. So when cyst was ruptured, ethanol exposure to near proximity and systemic absorption by vessels could cause drunken status and also neurodegenerative change in lumbar plexus. In our case, it was accidentally damaged femoral nerve.

Meralgia paresthetica was a differential diagnosis for our patient. The symptoms of lateral femoral cutaneous nerve entrapment, commonly known as “meralgia paresthetica,” include sensory loss, pain, and dysesthesia in part of the area supplied by the nerve. There is no motor component to the nerve, and therefore weakness is not a part of this syndrome. Symptoms often are worsened by standing or walking. Reflexes should remain intact. Our patient had pain, weakness, and paresthesia in anteromedial aspect of right thigh; also right knee deep tendon reflex (DTR) was absent, and so this differential diagnosis was ruled out for him. In conclusion, percutaneous renal cyst ethanol sclerotherapy appears to be not safe completely.

Femoral nerve injury caused by percutaneous simple renal cysts with ethanol is a very rare complication of the procedure. The main fault of the urologist who carried out the percutaneous procedure is to perform the procedure under only ultra sonography (US) guidance. Although the Akinci et al. study [11] in year 2005 suggested percutaneous alcohol sclerotherapy with needle aspiration or catheter drainage under US (preferable in cases of shallow and/or larger (>6 cm) cysts) or CT (suitable for treating deeper and/or smaller (<6 cm) cysts) guidance as an effective and safe method for treating symptomatic simple renal cysts, his study in year 2008 suggested catheter insertion for cysts larger than 4 cm in diameter under guidance of US and fluoroscopy [12]; so the authors believe that this procedure should be performed under US combined with fluoroscopic guidance in huge simple renal cyst for decrease in any similar complication.

To the best of our knowledge, this is the first report of femoral nerve injury caused by percutaneous simple renal cyst sclerotherapy with ethanol. This rare event has not been described previously; so physicians should be aware of the possibility of this complication. We suggest that it should be compared with another similar experience.
